# Inter-nostril olfactory asymmetry in nasal septal deviation: a controlled cross-sectional study

**DOI:** 10.3389/fsurg.2026.1961412

**Published:** 2026-10-30

**Authors:** Cemal Haci, Eren Yilmaz

**Affiliations:** Department of Otorhinolaryngology, Acibadem Taksim Hospital, Acibadem Healthcare Group, Istanbul, Türkiye

**Keywords:** monorhinal testing, nasal obstruction, nasal septal deviation, olfactory asymmetry, Sniffin' Sticks

## Abstract

**Introduction:**

Nasal septal deviation may be associated with side-specific differences in olfactory performance. This study aimed to compare side-specific olfactory function and absolute inter-nostril asymmetry between people with nasal septal deviation and healthy controls and to evaluate the association between the severity of global subjective nasal obstruction and directional olfactory asymmetry.

**Methods:**

A prospective single-center controlled cross-sectional study was conducted, including 61 patients with nasal septal deviation and 48 controls comparable in age and sex. Side-specific TDI scores (Sniffin' Sticks threshold, discrimination, and identification tests) for each nasal cavity were collected. The absolute inter-nostril asymmetry (|ΔTDI|) was calculated based on the side-to-side difference in TDI scores. Subjective nasal obstruction was assessed using a visual analog scale (VAS).

**Results:**

Composite TDI scores of patients with septal deviation were significantly lower on the deviated side than on the wider side (26.81 ± 5.49 vs. 31.77 ± 3.36, respectively; mean difference, 4.96 points; 95% confidence interval, 3.34–6.58; *p* < 0.001), whereas no significant side-to-side difference was found in controls (*p* = 0.236). Absolute inter-nostril asymmetry was significantly higher in patients than in controls (median [interquartile range] = 6.00 [2.25–9.50] vs. 2.13 [0.75–3.81]; *p* < 0.001). The correlation between the VAS score and directional olfactory asymmetry was positive in patients (Spearman's *ρ* = 0.561; *p* < 0.001).

**Conclusion:**

People with nasal septal deviation demonstrated decreased TDI scores on the deviated side and increased absolute inter-nostril asymmetry compared to controls. The clinical implications of these findings require further study.

## 1 Introduction

Nasal septal deviation (NSD) represents a common anatomical cause of nasal obstruction and has been found to be associated with changes in olfactory function (1, 2). However, the association between NSD and olfactory function remains controversial, as findings in the literature have varied because of heterogeneous study designs and olfactory assessment methods (3–5).

Existing studies often measure overall olfactory performance, which may mask differences in unilateral olfactory function in patients with asymmetrical pathology (6, 7). Experiments have shown that unilateral nostril olfactory source localization is different from bilateral nostril sampling; thus, unilateral olfactory assessment becomes particularly important (8). Due to the asymmetric nature of the pathology, olfactory performance in such patients could also be asymmetrical, with lower scores expected on the deviated side (4, 7). Indeed, Altundag et al. revealed that there were differences in olfactory function between the deviated and wider sides in patients with NSD (7). Fyrmpas et al. measured unilateral odor identification using a 12-item Sniffin' Sticks test before and after septoplasty and observed that patients with considerable inter-nostril differences in odor identification had more preoperative asymmetry in nasal airflow (9). Poupon et al. developed normative values and cut-off points for unilateral olfactory performance measured using the Sniffin' Sticks test in healthy individuals and hyposmic patients (10). Moreover, a systematic review and meta-analysis conducted by Grabosky et al. found modest improvement in olfactory function after septoplasty (11).

There could be some anatomical and flow characteristics—such as the geometric shape of the olfactory cleft, depth of olfactory sulcus, and local airflow—that influence odorant transport and olfactory perception (12–15). Psychophysical tests and consensus criteria are mandatory for the clinical diagnosis of olfactory dysfunction (16, 17). Taking into account the lateralized nature of olfactory function in patients with NSD revealed in previous studies, this study investigated the threshold, discrimination, and identification (TDI) scores of patients and the control group separately for each nostril. The contribution of this study is a controlled comparison of absolute inter-nostril TDI asymmetry between the two groups using the same measure. We also aimed to determine whether the severity of subjective obstruction in patients correlated with TDI asymmetry.

## 2 Materials and methods

### 2.1 Study design and participants

This prospective, single-center, controlled cross-sectional observational study took place in the Department of Otorhinolaryngology, Acibadem Taksim Hospital, Istanbul, Türkiye. Participants were enrolled from December 1, 2022, to December 1, 2024, using prospective clinical examinations and unilateral olfactory tests. Computed tomography (CT) images had been obtained for clinical indications, not for research purposes, and were available for all 61 patients with NSD; these images were not part of the prospective protocol and were analyzed retrospectively as a supplementary component. The study was approved by the Ethics Committee of Acibadem University (protocol code: 2022-17/40; date of approval: November 11, 2022) and was conducted according to the principles of the Declaration of Helsinki. The ethics approval covered the retrospective use of clinically acquired CT images for the supplementary anatomical assessment. Written informed consent, including consent for the retrospective use of clinically obtained imaging data, was obtained from all participants. The study was reported according to the STROBE guidelines for observational studies.

Patients aged between 18 and 40 years with complaints of nasal obstruction who were assessed at the outpatient clinic were screened for eligibility. Adults diagnosed with symptomatic NSD based on rigid nasal endoscopy were eligible for participation in the study. Individuals meeting the inclusion criteria and providing written informed consent constituted the NSD group.

The control group consisted of individuals aged between 18 and 40 years, recruited prospectively from patients visiting the otorhinolaryngology outpatient clinic for reasons other than nasal obstruction and smell loss, including cerumen impaction, itching of the ears, and otalgia caused by temporomandibular joint dysfunction. The controls underwent clinical assessment and rigid nasal endoscopy, and only those without clinically evident septal deviation or other nasoendoscopic abnormalities were enrolled in the study. In addition, the controls did not meet any of the predefined exclusion criteria. The controls were not matched with individual patients, but both groups were similar in terms of age and sex composition.

The exclusion criteria were set before recruitment and consistently used for both groups: chronic rhinosinusitis (with or without nasal polyps) (18), inferior turbinate hypertrophy, active allergic rhinitis, recent upper respiratory tract infection, previous nasal or sinus surgeries, current smoking, and any other neurological or neurodegenerative disorders affecting olfactory function. A recent upper respiratory tract infection was defined as an infection that occurred within four weeks before olfactory testing. Participants' medical history included questions about olfactory complaints after COVID-19 infection or other types of respiratory infections (19). Participants with persistent impairment or loss of olfactory function, or with parosmia, that appeared after infection were excluded from the study. This criterion was applied consistently for both patients and control participants to avoid possible bias due to acute or persistent postinfectious olfactory dysfunction (20). Only those participants who completed the examination and underwent olfactory testing on both sides were included in the analysis.

Of the 147 individuals screened (85 potential patients with NSD and 62 potential controls), 38 were excluded. In the NSD group, 24 individuals were excluded because of chronic rhinosinusitis (*n* = 5), nasal polyposis (*n* = 2), inferior turbinate hypertrophy (*n* = 6), active allergic rhinitis (*n* = 6), or previous nasal or sinus surgery (*n* = 5). In the control group, 14 individuals were excluded because of current smoking (*n* = 5), recent upper respiratory tract infection (*n* = 4), or inferior turbinate hypertrophy (*n* = 5). The final sample therefore comprised 109 participants: 61 patients with NSD and 48 controls (Supplementary Figure S1).

### 2.2 Nasal obstruction and olfactory assessment

All subjects underwent clinical assessment and rigid nasal endoscopy. Chronic rhinosinusitis was defined according to the EPOS 2020 criteria as two or more symptoms lasting ≥ 12 weeks, one of which had to be nasal blockage/obstruction/congestion or nasal discharge (anterior/posterior nasal drip), with or without facial pain/pressure or reduction/loss of smell, together with endoscopic evidence of nasal polyps, mucopurulent discharge, and/or edema/mucosal obstruction primarily in the middle meatus (18). Inferior turbinate hypertrophy was defined as an enlarged inferior turbinate in contact with the nasal septum or nasal floor on rigid endoscopy that did not shrink adequately after topical decongestion. Subjects meeting either definition, or showing other endoscopic signs of sinonasal inflammation, were excluded from the study. NSD was established using rigid nasal endoscopy. The side with more narrowing of the nasal cavity because of septal deviation was referred to as the deviated side, while the contralateral side was referred to as the wider side. In complex deviations, such as those subsequently classified as Mladina types 4 and 7 on CT, side assignment was based on the overall endoscopic assessment of nasal cavity narrowing attributable to septal deviation rather than on an isolated focal deformity. A single experienced endoscopist designated the deviated and wider sides before olfactory testing and independently of the subsequent retrospective CT assessment. The shape of the septum was classified according to the Mladina classification, and the degree of deviation was measured separately from the archival CT images of the patient group, as described in Section 2.3. There were no CT images available for the control group. Nasal obstruction was assessed subjectively using a 100-mm continuous visual analog scale (VAS); scores were expressed from 0 (absence of nasal obstruction) to 10 (total nasal obstruction).

Rigid nasal endoscopy and olfactory tests were performed by independent investigators. The investigators performing the olfactory tests were unaware of the endoscopic results and which side was deviated at the time of testing. No anesthetics or decongestants were used before the initial nasal endoscopy and olfactory test. Topical decongestant was applied only after completion of olfactory testing, after which the inferior turbinates were re-examined endoscopically to assess hypertrophy. Olfactory tests were performed during regular daytime hours in the outpatient clinics. Olfactory performance was evaluated using the standardized Sniffin’ Sticks test battery (Burghart Messtechnik, Wedel, Germany), which included odor threshold (T), odor discrimination (D), and odor identification (I). The first nostril tested was determined using a computer-generated randomization list, and then both nostrils were tested independently, with the contralateral nostril occluded with paper tape. The same odorants, order of presentation, and exposure time were used for both nostrils. Each odorant pen was presented for about 3 s. In the threshold and discrimination subtests, there was a break of about 3–5 s between presentations and of about 20–30 s between triplets. In the identification subtest, there was a pause of approximately 20–30 s between successive presentations. A 10-minute rest period was given between the tests of the two nostrils. The TDI score, which is the sum of T, D, and I scores, was calculated for each side. The scores were recorded for the right and left nostrils and, in the patient group, were later assigned to the deviated and wider sides.

### 2.3 Computed tomography-based anatomical and morphological assessment

CT scans of all 61 patients with NSD had been performed for clinical indications as part of routine evaluation, on the same day as olfactory testing. These images were analyzed retrospectively to classify septal morphology and measure the deviation angle. Both assessments were performed independently by two observers who were blinded to the patients' clinical data, endoscopic side assignment, VAS scores, and olfactory test results. For the Mladina classification, inter-rater agreement was assessed using the initial independent ratings, before consensus, with unweighted Cohen's kappa (*κ*), and disagreements were resolved by consensus to determine the final type. For the deviation angle, the mean of the two independent measurements was used as the final value, and inter-rater reliability was evaluated using a two-way random-effects, absolute-agreement, average-measures intraclass correlation coefficient (ICC) with 95% confidence intervals.

• Mladina Morphology Types: Coronal and axial views were assessed simultaneously to categorize individuals into Mladina types 1–7 (21). Although the Mladina system primarily describes the shape of the deformity rather than its precise septal area, types 1 and 2 broadly correspond to anterior deviations in the nasal valve area, type 3 to a deviation at the level of the middle turbinate head, type 4 to an S-shaped deviation involving both areas, types 5 and 6 to horizontal crests or spurs along the septal base, and type 7 to combined deformities (22).

• Deviation Angle Analysis: The deviation angle was measured in degrees (°) using coronal multiplanar reconstruction CT images after correction for head rotation and tilt. The coronal slice demonstrating the largest deviation angle was selected for measurement. A reference midline was drawn from the crista galli–septum junction to the midline of the nasal floor, and a second line was drawn from the crista galli–septum junction to the apex of the septal deviation; the angle between these two lines was recorded as the deviation angle. In cases with bony spurs, the apex was defined on the main structural contour of the septum rather than at the tip of the localized spur. In S-shaped (Mladina type 4) deviations, the angle was measured separately for each curve, and the larger value was used for analysis. Deviation severity was classified as mild (< 9°), moderate (9°–15°), or severe (> 15°) (23).

### 2.4 Outcome definitions

Directional olfactory asymmetry was evaluated in the patient group as the TDI score on the wider side minus the TDI score on the deviated side.


ΔTDI=TDI_wider−TDI_deviated

Positive values indicate relatively lower olfactory performance on the deviated side.

To enable a direct comparison between patients and controls using a consistent, direction-independent measure, absolute inter-nostril asymmetry (|ΔTDI|) was calculated for both groups using side-specific composite scores:

$$\begin{array}{c} \mathrm{In}\;\mathrm{patients}:\;|\Delta \mathrm{TDI}|=|\mathrm{TDI}\_\mathrm{wider}-\mathrm{TDI}\_\mathrm{deviated}|\\ \mathrm{In}\;\mathrm{controls}:\;|\Delta \mathrm{TDI}|=|\mathrm{TDI}\_\mathrm{right}-\mathrm{TDI}\_\mathrm{left}| \end{array}$$

Because absolute differences eliminate the directional sign, both formulations represent the mathematically equivalent, direction-independent magnitude of inter-nostril asymmetry.

Published Turkish and European normative data were used as contextual references for TDI scores rather than as diagnostic cut-offs for individual nostrils (24, 25).

The threshold of |ΔTDI| ≥ 3 points was established *a priori* for exploratory purposes. This value was extrapolated from previous literature, specifically Fyrmpas et al. (9), who utilized a 3-point asymmetry cut-off for the 12-item identification test. We applied this same numerical threshold to the composite TDI score as an exploratory baseline to investigate whether this previously reported inter-nostril difference yields meaningful clinical separation across the comprehensive test battery. This threshold is not intended as an established diagnostic threshold for any clinical impairment of the sense of smell. The main results were based on comparisons of continuous TDI scores and absolute inter-nostril differences.

### 2.5 Statistical analysis

An *a priori* sample size calculation was performed before the start of data collection using G*Power version 3.1.9.7 (Heinrich Heine University Düsseldorf, Düsseldorf, Germany). The primary outcome was the within-patient difference in TDI scores between the wider and deviated nasal cavities. The expected effect size was estimated from preliminary pilot measurements by dividing the mean paired TDI difference by the standard deviation of the paired differences. To avoid bias and data contamination, the 10 participants in the preliminary pilot phase were kept entirely independent of the final study sample and were not included in the present analyses. Using a two-sided paired-samples t-test, an alpha level of 0.05, a target power of 95%, and an estimated effect size of dz = 0.90, the minimum required sample was 19 patients. Recruitment continued throughout the predefined study period, resulting in the enrollment of 61 patients with NSD and 48 controls. Between-group comparisons of absolute inter-nostril asymmetry were additional analyses, whereas the |ΔTDI| ≥ 3 threshold analysis was secondary and exploratory.

Statistical analyses were performed using IBM SPSS Statistics for Windows, Version 26.0 (IBM Corp., Armonk, NY, USA). Continuous variables were evaluated for normality using the Shapiro–Wilk test. Normally distributed variables are presented as mean ± standard deviation (SD), and non-normally distributed variables are presented as median and interquartile range (IQR). Categorical variables are presented as frequencies and percentages.

Age and sex distributions between groups were compared using an independent-samples t-test and Pearson's chi-square test, respectively. VAS scores and absolute inter-nostril TDI differences (|ΔTDI|) were compared between groups using the Mann–Whitney U test. For descriptive purposes, mean group differences for non-normally distributed continuous variables were also reported with 95% confidence intervals estimated using bootstrap methods. Within-group comparisons of side-specific TDI and subtest scores were performed using paired-samples t-tests or Wilcoxon signed-rank tests, as appropriate. The Holm–Bonferroni procedure was used to adjust *p* values for the three subtest comparisons (T, D, and I) within each group. To reduce potential confounding, enrollment was restricted to adults aged 18–40 years, and the same predefined exclusion criteria were applied to both groups. No multivariable adjustment for age, sex, or other potential confounders was performed. Between-group comparisons and analyses of the association between VAS and directional ΔTDI were therefore unadjusted.

The proportions of participants meeting the exploratory |ΔTDI| ≥ 3 threshold were compared using Pearson's chi-square test, with odds ratios (OR) and 95% confidence intervals (CI) reported. Additional *post hoc* sensitivity analyses used alternative exploratory thresholds of |ΔTDI| ≥ 2 and ≥4 points, applying the same statistical approach.

An additional *post hoc* sensitivity analysis was performed to assess whether side assignment in complex septal deviations influenced the main findings. For this purpose, patients classified on CT as Mladina type 4 (S-shaped deviation) or type 7 (combined deformities) (*n* = 15) were excluded, leaving 46 patients with non-complex deviations, who were compared with the 48 controls. In this subset, the normality of paired TDI differences was re-evaluated using the Shapiro–Wilk test, and within-patient TDI differences between the deviated and wider sides were reanalyzed using a paired-samples t-test or Wilcoxon signed-rank test, as appropriate. |ΔTDI| was compared between groups using the Mann–Whitney U test.

The association between the VAS scores and directional ΔTDI was assessed using Spearman's rank correlation. A simple linear regression model was also fitted, with the VAS score as the predictor and directional ΔTDI as the outcome. Linearity, normality of residuals, and homoscedasticity were assessed using diagnostic plots. The unstandardized regression coefficient (B) and coefficient of determination (R^2^) are reported. As a supplementary exploratory analysis, the association between the CT-measured deviation angle and directional ΔTDI was assessed using Spearman's rank correlation. Inter-rater agreement for the CT-based assessments was analyzed using Cohen's kappa and the ICC, as described in Section 2.3.

All tests were two-sided, and *p* < 0.05 was considered statistically significant.

## 3 Results

### 3.1 Participants and group characteristics

Of the 147 individuals assessed for eligibility, 38 did not meet the eligibility criteria and were excluded, leaving 109 participants (61 patients with NSD and 48 controls; Supplementary Figure S1). There were no missing data for any analyzed variable in either group. The groups were similar in mean age (±SD) (28.98 ± 4.27 vs. 29.27 ± 4.81 years; *p* = 0.742) and sex distribution (females/males: 28/33 vs. 22/26, respectively; *p* = 0.994). The median VAS score for nasal obstruction was higher in the patients than in the controls (median [IQR], 6.0 [5.0–7.5] vs. 2.0 [1.0–2.0]; *p* < 0.001) (Table 1).

**Table 1:** Demographic and clinical characteristics.

Variable	Septal Deviation (*n* = 61)	Control (*n* = 48)	*p* value
Age (years), mean ± SD	28.98 ± 4.27	29.27 ± 4.81	0.742^a^
Sex (Female/Male), n	28/33	22/26	0.994^b^
VAS, median [IQR]	6.0 [5.0–7.5]	2.0 [1.0–2.0]	<0.001^c^

Data are presented as mean ± SD, median [IQR], or n. IQR, interquartile range; SD, standard deviation; VAS, visual analog scale.

a Independent-samples t-test;

b Pearson's chi-square test;

c Mann–Whitney U test.

The additional retrospective review of archival CT images included all 61 patients with NSD. Based on the endoscopic side assignment, deviation was on the right side in 25 patients (41.0%) and on the left in 36 (59.0%). The most common Mladina types were type 3 (*n* = 16, 26.2%) and type 2 (*n* = 14, 23.0%), followed by type 4 (*n* = 12, 19.7%), type 5 (*n* = 6, 9.8%), type 1 (*n* = 5, 8.2%), type 6 (*n* = 5, 8.2%), and type 7 (*n* = 3, 4.9%). Inter-rater agreement for the Mladina classification was *κ* = 0.84 (95% CI, 0.76–0.92), and the average-measures ICC for deviation-angle measurements was 0.91 (95% CI, 0.86–0.95). The mean deviation angle was 12.84 ± 3.42°. According to the deviation-severity classification described in the Methods section, 12 patients (19.7%) showed mild deviation (<9°), 31 (50.8%) had moderate deviation (9°–15°), and 18 (29.5%) presented with severe deviation (>15°). In the additional exploratory analysis, increased deviation angles were significantly correlated with greater directional olfactory asymmetry (ΔTDI; Spearman's *ρ* = 0.482; *p* < 0.001).

### 3.2 Normality and within-group side-specific comparisons

Paired differences for TDI and T were normally distributed (Shapiro–Wilk *p* ≥ 0.130). Thus, paired t-tests were used for these comparisons. Conversely, paired side-to-side differences in discrimination and identification scores were not normally distributed; hence, non-parametric tests were used.

For the NSD group, the composite TDI score of the deviated side was significantly lower than that of the wider side (26.81 ± 5.49 vs. 31.77 ± 3.36; mean difference = 4.96; SD of difference = 6.32; 95% CI = 3.34–6.58; *p* < 0.001). Similarly, the threshold, discrimination, and identification scores were significantly lower on the deviated side (all *p* < 0.001).

In contrast, the control group showed no statistically significant side-to-side differences in the composite TDI score (31.74 ± 3.27 vs. 31.19 ± 3.22; *p* = 0.236) or in the T, D, and I subtest scores (*p* ≥ 0.615) (Table 2; Figures 1A,B).

**Table 2:** Side-specific Sniffin’ Sticks subtest and composite TDI comparisons.

A. Patients with nasal septal deviation (*n* = 61)			
Outcome	Deviated side	Wider side	*p* value
T	6.41 ± 1.55	7.87 ± 1.29	<0.001^a^^,^^c^
D	10.02 ± 2.09 (10.0 [9.0–11.0])	11.64 ± 1.38 (11.0 [11.0–12.0])	<0.001^b^^,^^c^
I	10.38 ± 2.32 (11.0 [9.0–12.0])	12.26 ± 1.35 (12.0 [12.0–13.0])	<0.001^b^^,^^c^
TDI	26.81 ± 5.49	31.77 ± 3.36	<0.001^a^

B. Controls (*n* = 48)			
Outcome	Right nostril	Left nostril	*p* value
T	7.68 ± 0.95	7.52 ± 1.00	0.621^a^^,^^c^
D	11.77 ± 1.19 (12.0 [11.0–12.75])	11.54 ± 1.41 (12.0 [11.0–12.0])	0.615^b^^,^^c^
I	12.29 ± 1.57 (12.0 [11.0–13.0])	12.13 ± 1.44 (12.0 [11.0–13.0])	0.638^b^^,^^c^
TDI	31.74 ± 3.27	31.19 ± 3.22	0.236^a^

Values are presented as mean ± standard deviation; for D and I, median [interquartile range] is also shown in parentheses. T, odor threshold; D, odor discrimination; I, odor identification; TDI, composite threshold–discrimination–identification score.

a Paired-samples t-test.

b Wilcoxon signed-rank test.

c *p* values adjusted using the Holm–Bonferroni procedure for the three subtest comparisons within each group.

**Figure 1:**
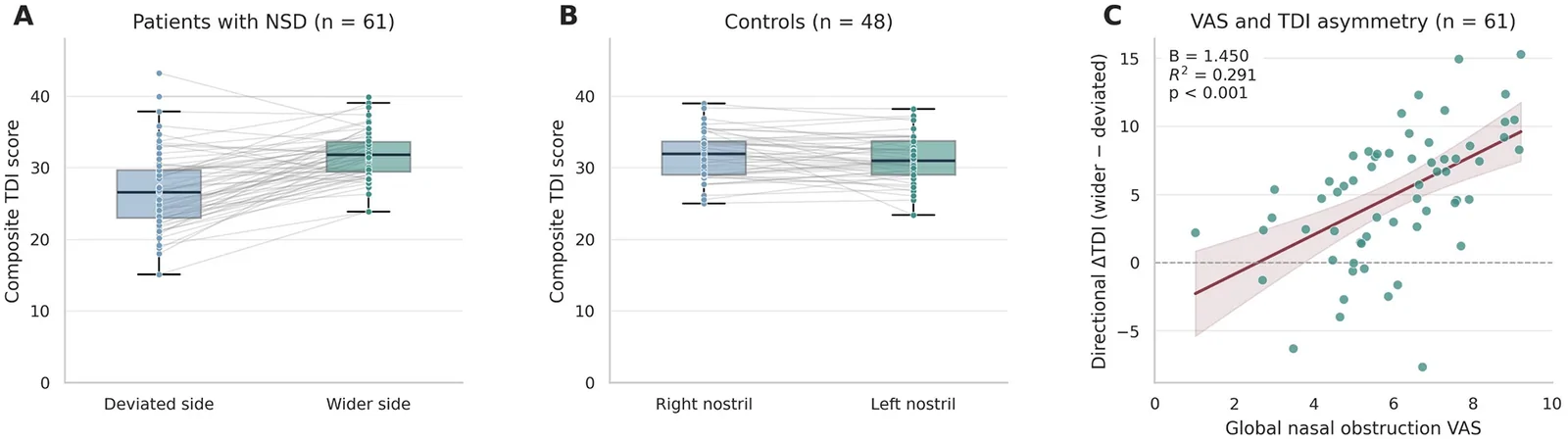
Side-specific olfactory evaluation and inter-nostril asymmetry in patients with nasal septal deviation and control participants. **(A)** Individual paired composite TDI scores on the deviated versus wider nasal cavities in patients with nasal septal deviation (*n* = 61), demonstrating significantly lower olfactory performance on the deviated side (*p* < 0.001, paired t-test). **(B)** Composite TDI scores for the right and left nasal cavities in control participants (*n* = 48), showing no significant side-to-side differences (*p* = 0.236, paired t-test). Boxes indicate the median and interquartile range (IQR); whiskers extend to 1.5 × IQR. **(C)** Scatter plot and linear regression model illustrating the positive association between global visual analog scale (VAS) nasal obstruction scores and directional olfactory asymmetry (ΔTDI = TDI_wider−TDI_deviated) in patients (R^2^ = 0.291, B = 1.450, *p* < 0.001). The solid line represents the fitted regression line with its 95% confidence interval (shaded area); the dashed line indicates ΔTDI = 0 (no asymmetry), and values below it reflect higher scores on the deviated side.

### 3.3 Between-group asymmetry and association with VAS

Since the |ΔTDI| scores were not normally distributed in either group, they are presented as medians and interquartile ranges. The median |ΔTDI| score was significantly higher in patients with NSD than in the control subjects (6.00 [2.25–9.50] vs. 2.13 [0.75–3.81], respectively; *p* < 0.001). An absolute inter-nostril TDI difference of ≥3 points was found in 40 patients (65.6%) and 20 control subjects (41.7%) (odds ratio = 2.67; 95% CI, 1.23–5.79; *p* = 0.013) (Table 3).

**Table 3:** Absolute inter-nostril TDI asymmetry between groups.

Outcome	Septal deviation (*n* = 61)	Control (*n* = 48)	Group difference/effect (95% CI)	*p* value
Absolute |ΔTDI|, median [IQR]	6.00 [2.25–9.50]	2.13 [0.75–3.81]	–	<0.001^a^
Absolute |ΔTDI|, mean ± SD	6.43 ± 4.78	2.49 ± 2.08	Mean difference: 3.94 (2.59–5.30)	–
Post hoc sensitivity |ΔTDI| ≥ 2, *n* (%)	54 (88.5%)	28 (58.3%)	OR: 5.51 (2.08–14.60)	<0.001^b^
Exploratory |ΔTDI| ≥ 3, *n* (%)	40 (65.6%)	20 (41.7%)	OR: 2.67 (1.23–5.79)	0.013^b^
Post hoc sensitivity |ΔTDI| ≥ 4, *n* (%)	38 (62.3%)	12 (25.0%)	OR: 4.96 (2.16–11.38)	<0.001^b^

Mean difference is presented with bootstrap 95% CI. CI, confidence interval; IQR, interquartile range; OR, odds ratio; SD, standard deviation; TDI, threshold–discrimination–identification score.

a Mann–Whitney U test;

b Pearson's chi-square test.

Furthermore, in *post hoc* sensitivity analyses, a difference of at least 2 points in the TDI score between the nostrils was found in 54 patients (88.5%) and 28 control subjects (58.3%), with an OR of 5.51 (95% CI, 2.08–14.60; *p* < 0.001). A difference of ≥4 points was found in 38 patients (62.3%) and 12 control subjects (25.0%), with an OR of 4.96 (95% CI, 2.16–11.38; *p* < 0.001).

In the *post hoc* sensitivity analysis excluding patients with Mladina type 4 or 7 deviations (*n* = 15), the paired TDI differences remained normally distributed (Shapiro–Wilk *p* = 0.284). For the remaining 46 patients, the composite TDI score was still significantly lower on the deviated side than on the wider side (26.93 ± 5.32 vs. 31.82 ± 3.28; mean difference = 4.89; SD of difference = 6.10; 95% CI = 3.08–6.70; paired-samples t-test, *p* < 0.001). Furthermore, the median |ΔTDI| remained significantly higher in these 46 patients than in the 48 controls (5.75 [2.25–9.00] vs. 2.13 [0.75–3.81]; Mann–Whitney U test, *p* < 0.001). Thus, excluding complex deviations did not change the direction or statistical significance of the main findings.

In this patient sample, higher global subjective nasal obstruction ratings on the VAS were associated with greater directional olfactory asymmetry (Spearman's *ρ* = 0.561; *p* < 0.001). In simple linear regression, each one-unit increase in VAS score was associated with a 1.45-unit increase in directional ΔTDI (B = 1.450; 95% CI, 0.860–2.040; *p* < 0.001). The VAS score explained 29% of the variance in directional asymmetry (R^2^ = 0.291) (Figure 1C).

## 4 Discussion

The TDI scores for patients with NSD were lower on the deviated side than on the wider side, with a mean TDI difference of 4.96, although approximately one-fifth of the patients (21%) showed equal or higher scores on the deviated side (Figures 1A,C). In contrast, the control group demonstrated no significant difference between the two sides. The absolute inter-nostril TDI asymmetry in patients was higher than that in the control group. These findings indicate an association between the two factors; however, because of the study's observational nature, no cause-and-effect relationship can be established.

Earlier studies have shown lateralization of olfactory function in patients with nasal septal deviation. The results of the current study support the finding of poorer olfaction on the deviated side reported by Altundag et al. (7). Fyrmpas et al. studied odor identification with a 12-item Sniffin' Sticks test and found greater nasal flow differences preoperatively among patients with greater differences in inter-nostril odor identification ability (9). However, the reduction in the postoperative prevalence of such patients did not reach statistical significance. In our study, we examined all three aspects of olfaction using the total TDI score and compared absolute inter-nostril asymmetry between patients and controls in a direction-independent manner.

Poupon et al. also provided specific normative data for monorhinal testing (10), which offer more guidance on the interpretation of nostril-specific scores than generic birhinal norms. In the current study, the side-to-side score difference was used as an indicator of relative differences in olfactory ability, without assigning nostril-specific diagnostic categories. Concerning surgical outcomes, Grabosky et al. observed modest improvements in test scores after septoplasty in a meta-analysis (11), and Valsamidis et al. similarly evaluated olfactory outcomes after septoplasty in patients with NSD (26). Directional ΔTDI was used to estimate whether olfactory performance was decreased on the deviated side, whereas absolute inter-nostril asymmetry (|ΔTDI|) enabled assessment of the degree of asymmetry in patients and controls regardless of direction. In the additional exploratory analysis, |ΔTDI| ≥ 3 was more common in patients than in controls (65.6% vs. 41.7%). However, the three-point difference used by Fyrmpas et al. (9) for the 12-item identification test has not been validated for the total TDI score, and its presence in 41.7% of controls further limits the diagnostic interpretation of such results. Therefore, the main interpretation is based on continuous TDI comparisons; further validation and retesting are necessary before this exploratory cut-off can be used.

Higher global VAS scores for subjective nasal obstruction were moderately associated with greater directional TDI asymmetry, with the regression analysis explaining 29% of the variance. Because the global VAS measured overall nasal obstruction without distinguishing between the two nasal cavities, this finding indicates an association between the severity of subjective symptoms and the inter-nostril difference in olfactory function. This association does not indicate a direct link between ipsilateral obstruction and decreased olfactory function. Notably, the control group reported low but non-zero VAS scores (median 2.0 [IQR 1.0–2.0]), even in the absence of clinically important septal deviation or any other endonasal pathology confirmed by clinical examination and nasal endoscopy. In addition, the exploratory supplementary analysis using retrospective CT imaging showed a positive correlation between the deviation angle and TDI asymmetry (Spearman's *ρ* = 0.482; *p* < 0.001). This association indicates a possible link between the greater deviation angle and inter-nostril asymmetry of olfactory function.

Deviation of the septum may influence local flow dynamics and reduce the delivery of odorants to the olfactory cleft. The efficiency of the olfactory system may be additionally influenced by the shape of the olfactory cleft, the anatomical features of the olfactory sulcus, and regional airflow (12–15, 27). This might explain the low TDI values on the deviated side. However, this proposed mechanism could not be directly tested in the present study.

The present findings indicate differences in olfactory function between the nasal passages. Because birhinal olfactory testing was not performed, this study cannot determine whether patients with NSD had inferior olfactory function compared with those without NSD, or whether unilateral testing improves treatment decisions or predicts postoperative olfactory benefit.

This study has several limitations. It included only adult patients (18–40 years old) from a single center. Although the groups were similar in age and sex, this does not exclude confounding. Because no multivariable adjustment was performed, residual confounding may have influenced the between-group comparisons and the association between VAS and directional ΔTDI. Retrospectively retrieved archival CT images allowed septal deviation to be classified and its degree to be measured; however, these images provide only static structural information. Objective measurements of nasal airflow and visualization of the olfactory cleft were not performed, so the proposed airflow-related mechanism could not be tested. In addition, deviation was characterized by Mladina type and deviation angle, which describe the shape and severity of the deformity but not its precise location along the septum (anterior/valve area versus posterior); therefore, the relationship between deviation site and olfactory asymmetry could not be assessed. Furthermore, the VAS was used to assess the overall degree of nasal obstruction without taking side-specific symptoms into account. Consequently, the relationship between side-specific subjective nasal obstruction and ipsilateral olfactory performance could not be assessed. The nasal cycle was not controlled, and olfactory testing was performed only once, which might have contributed to inter-nostril variability. Although the first tested side was randomized and a rest interval was provided between tests, an order effect cannot be completely ruled out. Postoperative follow-up was not performed. Further multicenter and longitudinal research with objective airflow measurements and repeated olfactory tests will help to confirm these findings and evaluate post-septoplasty changes (26, 28).

## 5 Conclusions

NSD was associated with reduced TDI scores on the deviated side and with increased absolute inter-nostril olfactory asymmetry compared with control subjects. In patients, the global subjective severity of nasal obstruction was moderately associated with a larger TDI difference between the wider and deviated nasal passages. The significance of inter-nostril differences for treatment purposes remains unknown. The |ΔTDI| ≥ 3 threshold should be considered preliminary rather than diagnostic. Additional research with objective measurements and follow-up is required. Because birhinal olfactory testing was not performed and no postoperative outcomes were assessed, these findings do not permit conclusions regarding overall olfactory loss or surgical success.

## Data Availability

The raw data supporting the conclusions of this article will be made available by the authors, without undue reservation.

## Supplementary material

Supplementary Figure S1 Participant selection and reasons for exclusion. Flow diagram showing the number of individuals assessed for eligibility, excluded (with reasons, reported separately for patients with nasal septal deviation and controls), and included in the analysis. NSD, nasal septal deviation.
