# Supplementary material for: Inter-nostril olfactory asymmetry in nasal septal deviation: a controlled cross-sectional study
Source: Front Surg. 2026 Oct 30;13:1961412. doi: 10.3389/fsurg.2026.1961412 (PMC13759846; doi:10.3389/fsurg.2026.1961412)
Supplement: Supplementary file 1 [file Table1.docx]

STROBE checklist

**Inter-Nostril Olfactory Asymmetry in Nasal Septal Deviation: A Controlled Cross-Sectional Study**

Cross-sectional study. Locations refer to manuscript sections, tables and supplementary material.

| **Section / topic** | **Item** | **Recommendation** | **Location / comment** |
| --- | --- | --- | --- |
| **Title and abstract** | | | |
| Title and abstract | 1a | Indicate the study's design with a commonly used term in the title or the abstract | Title; Abstract, Methods. |
|  | 1b | Provide an informative and balanced summary of what was done and what was found | Abstract. |
| **Introduction** | | | |
| Background/rationale | 2 | Explain the scientific background and rationale for the investigation | Introduction, paragraphs 1–3. |
| Objectives | 3 | State specific objectives, including any prespecified hypotheses | Introduction, final paragraph. |
| **Methods** | | | |
| Study design | 4 | Present key elements of study design early in the paper | Section 2.1, first paragraph. |
| Setting | 5 | Describe the setting, locations, and relevant dates, including periods of recruitment, exposure, follow-up, and data collection | Sections 2.1 and 2.3. |
| Participants | 6 | Give the eligibility criteria, and the sources and methods of selection of participants | Section 2.1. |
| Variables | 7 | Clearly define all outcomes, exposures, predictors, potential confounders, and effect modifiers. Give diagnostic criteria, if applicable | Sections 2.1–2.4. |
| Data sources / measurement | 8* | For each variable of interest, give sources of data and details of methods of assessment. Describe comparability of assessment methods if there is more than one group | Sections 2.2 and 2.3. |
| Bias | 9 | Describe any efforts to address potential sources of bias | Sections 2.1–2.3. |
| Study size | 10 | Explain how the study size was arrived at | Section 2.5, first paragraph. |
| Quantitative variables | 11 | Explain how quantitative variables were handled in the analyses. If applicable, describe which groupings were chosen and why | Sections 2.3–2.5. |

| **Section / topic** | **Item** | **Recommendation** | **Location / comment** |
| --- | --- | --- | --- |
| **Methods continued** | | | |
| Statistical methods | 12a | Describe all statistical methods, including those used to control for confounding | Section 2.5. No multivariable adjustment was performed. |
|  | 12b | Describe any methods used to examine subgroups and interactions | No subgroup or interaction analyses were performed. The analysis excluding complex deviations (Mladina types 4 and 7) is a sensitivity analysis; see item 12e. |
|  | 12c | Explain how missing data were addressed | Section 2.1 - 3.1: only participants who completed bilateral olfactory testing were included. There were no missing data for the analysed variables; therefore, no imputation was required. |
|  | 12d | If applicable, describe analytical methods taking account of sampling strategy | Not applicable. |
|  | 12e | Describe any sensitivity analyses | Section 2.5: alternative asymmetry thresholds and exclusion of complex deviations. |
| **Results** | | | |
| Participants | 13a* | Report numbers of individuals at each stage of study — e.g., numbers potentially eligible, examined for eligibility, confirmed eligible, included in the study, and analysed | Sections 2.1 and 3.1; Supplementary Figure S1 (NSD: 85 assessed, 61 analysed; controls: 62 assessed, 48 analysed). |
|  | 13b* | Give reasons for non-participation at each stage | Section 2.1; Supplementary Figure S1 (reasons for exclusion reported separately for each group). |
|  | 13c* | Consider use of a flow diagram | Supplementary Figure S1. |
| Descriptive data | 14a* | Give characteristics of study participants (e.g., demographic, clinical, social) and information on exposures and potential confounders | Section 3.1; Table 1. |
|  | 14b* | Indicate number of participants with missing data for each variable of interest | No missing data for any variable of interest in either group (Section 3.1). |
| Outcome data | 15* | Report numbers of outcome events or summary measures | Sections 3.2 and 3.3; Tables 2 and 3. |

| **Section / topic** | **Item** | **Recommendation** | **Location / comment** |
| --- | --- | --- | --- |
| **Results continued** | | | |
| Main results | 16a | Give unadjusted estimates and, if applicable, confounder-adjusted estimates and their precision (e.g., 95% CI). Make clear which confounders were adjusted for and why | Sections 3.2 and 3.3; Tables 2 and 3; Figure 1C. Estimates are unadjusted; groups were comparable in age and sex (Table 1). |
|  | 16b | Report category boundaries when continuous variables were categorized | Sections 2.3–2.5, 3.1 and 3.3; Table 3. |
|  | 16c | If relevant, consider translating estimates of relative risk into absolute risk for a meaningful time period | Not applicable to this cross-sectional study. |
| Other analyses | 17 | Report other analyses done — e.g., analyses of subgroups and interactions, and sensitivity analyses | Section 3.1 (exploratory CT deviation-angle analysis); Section 3.3 (post hoc sensitivity analyses: alternative thresholds and exclusion of Mladina types 4 and 7); Table 3. |
| **Discussion** | | | |
| Key results | 18 | Summarise key results with reference to study objectives | Discussion, first paragraph. |
| Limitations | 19 | Discuss limitations of the study, taking into account sources of potential bias or imprecision. Discuss both direction and magnitude of any potential bias | Discussion, final paragraph. |
| Interpretation | 20 | Give a cautious overall interpretation of results considering objectives, limitations, multiplicity of analyses, results from similar studies, and other relevant evidence | Discussion; Conclusions. |
| Generalisability | 21 | Discuss the generalisability (external validity) of the study results | Discussion, final paragraph. |
| **Other information** | | | |
| Funding | 22 | Give the source of funding and the role of the funders for the present study and, if applicable, for the original study on which the present article is based | Funding. No external funding. |

* Report information separately for patients with nasal septal deviation and controls.
